# *Schistosoma*, other helminth infections, and associated risk factors in preschool-aged children in urban Tanzania

**DOI:** 10.1371/journal.pntd.0006017

**Published:** 2017-11-06

**Authors:** Khadija Said, Jerry Hella, Stefanie Knopp, Tatu Nassoro, Neema Shija, Fatma Aziz, Francis Mhimbira, Christian Schindler, Upendo Mwingira, Anna M. Mandalakas, Karim Manji, Marcel Tanner, Jürg Utzinger, Lukas Fenner

**Affiliations:** 1 Ifakara Health Institute, Bagamoyo Research and Training Centre, Bagamoyo, Tanzania; 2 Swiss Tropical and Public Health Institute, Basel, Switzerland; 3 University of Basel, Basel, Switzerland; 4 Temeke Municipal Council Hospital, Dar es Salaam, Tanzania; 5 Neglected Tropical Disease Programme, Dar es Salaam, Tanzania; 6 National Institute for Medical Research, Dar es Salaam, Tanzania; 7 The Global Tuberculosis Program, Texas Children’s Hospital, Baylor College of Medicine, Houston, United States of America; 8 Muhimbili University of Health and Allied Sciences, Dar es Salaam, Tanzania; 9 Institute of Social and Preventive Medicine, University of Bern, Bern, Switzerland; Weill Cornell Medical College, UNITED STATES

## Abstract

**Background:**

Despite the high prevalence of helminth infections among preschool-aged children, control programs in sub-Saharan countries primarily focus on school-aged populations. We assessed the prevalence of helminth infections and determined risk factors for infection among preschool-aged children in the urban setting of Dar es Salaam, Tanzania.

**Methodology:**

Starting in October 2015, we conducted a 12-month prospective study among tuberculosis (TB)-exposed children under the age of 5 years and unexposed controls from neighboring households. At the time of recruitment, we collected medical histories, assessed development and cognitive functions, and performed medical examinations. We performed full blood cell counts and screened for HIV and malaria. Point-of-care circulating cathodic antigen (POC-CCA), urine filtration, Kato-Katz, FLOTAC, and Baermann tests were employed to detect helminth infections in urine and stool. Helminth infections were stratified for *Schistosoma* and other helminths to identify risk factors, using logistic regression.

**Principal findings:**

We included 310 children with a median age of 26 months (inter quartile range 17–42 months) in the study. Among these, 189 were TB-exposed and 121 TB-unexposed. Two thirds of the children were anemic (hemoglobin level <11 g/dl) and the HIV prevalence was 1.3%. *Schistosoma* spp. was the predominant helminth species (15.8%; 95% confidence interval [CI] 12.1–20.3%). Other helminth infections were less frequent (9.0%, 95% CI 6.3–12.8%). Poor hygiene, use of household water sources, and TB-exposure were not associated with helminth infection. Development and cognitive scores did not significantly differ in helminth-infected and uninfected peers, but hemoglobin levels were significantly lower in helminth-infected children (10.1 g/dl *vs*. 10.4 g/dl, p = 0.027).

**Conclusions/significance:**

In Dar es Salaam, a city with more than 4 million inhabitants, the prevalence of *Schistosoma* spp. infection among preschool-aged children was unexpectedly high. Setting-specific interventions that target preschool-aged children and urban settlements should be considered to reduce the transmission of *Schistosoma* and other helminth infections and to improve children’s health.

## Author summary

In many African countries, children under the age of 5 years are at considerable risk of acquiring parasitic worm infections. Yet, most of the neglected tropical disease control programs in Africa do not include preschool-aged children in deworming campaigns. Chronic parasitic worm infections may impair children’s growth and their cognitive development. We conducted a 12-month prospective study of children younger than 5 years in the Temeke district, Dar es Salaam—the economic capital of Tanzania—to assess the prevalence of parasitic worm infections. Among 310 included children, we found that one in six children was infected with the blood fluke *Schistosoma*, while one in 11 children were infected with soil-transmitted helminths. Anemia was found among 65% of children, particularly among those infected with parasitic worms. The high prevalence of *Schistosoma* infection in this urban setting, despite improved water supply and sanitation as well as limited open freshwater contact shows the pressing need to identify parasitic worm transmission hotspots in urban areas. Setting-specific interventions targeting preschool-aged children and urban settlements, among others, should be considered to reduce the transmission of *Schistosoma* and other parasitic worm infections.

## Introduction

Helminth infections affect more than 1.5 billion people globally and are particularly common amongst economically deprived populations [1, 2]. The burden of helminthiases is high in settings with inadequate sanitation, overcrowding, and low socioeconomic status; the same characteristics that govern transmission of tuberculosis (TB) [3–7]. Helminth infections, though rarely fatal, cause considerable morbidity [8, 9]. In children, heavy intensity helminth infections can impair physical growth and cognitive development, and lead to micronutrient deficiencies and anemia [3, 10]. Subsequently, if anemia and its underlying causes are not managed, it may lead to death in children with additional co-morbidities [11, 12]. Children with poor cognitive development have difficulties learning and perform poorly at school, thereby failing to reach their full potential [13]. Chronic helminth infection is also detrimental to the functioning of the immune response against infectious diseases such as TB and, hence, increases the risk of developing TB in later life [14]. Associations between TB and helminth infections have been reported for school-aged and adult populations [6, 15].

Children living in resource-constrained areas in sub-Saharan Africa and elsewhere are at high risk of acquiring helminth infections, given their poor hygienic environments and unattended outdoor access when playing with peers. Early detection and effective management of helminth infection can improve children’s health and wellbeing. Most studies of helminth infections have focused on school-aged populations, though preschool-aged children in highly endemic areas might also show high infection rates [16]. For example, a community-based, cross-sectional survey conducted in Nairobi found that the soil-transmitted helminth prevalence among preschool-aged children was similar to that of school-aged children [17]. In 2008, the World Health Organization (WHO) set an ambitious goal to reach 100% anthelmintic drug coverage by 2012 in endemic countries [18]. Yet, the WHO did not include preschool-aged children in targeted deworming campaigns until 2008.

In 2009, Tanzania adopted the WHO initiative to integrate preventive chemotherapy into its neglected tropical diseases control program, which also covers helminthiases. To date, the focus has been on school-aged children and adults [19]. No universal guidelines exist for using chemotherapy to prevent various helminth infections in preschoolers. To assess the prevalence and intensity of helminth infections among preschool-aged children, including its impact on clinical outcomes, we conducted a cross-sectional survey in an urban setting in Temeke district, Dar es Salaam, Tanzania. We employed a suite of standardized, quality-controlled diagnostic methods to enhance the accuracy of species-specific helminth detection and quantification [20].

## Methods

### Ethics statement

The study was approved by the Institutional Review Board of the Ifakara Health Institute (reference no. IHI/IRB 12–2015), the Medical Research Coordinating Committee of the National Institute of Medical Research in Tanzania (reference no. NIMR/HQ/R.8a/Vol. IX/2002), and the Ethics Committee of Northwestern and Central Switzerland (reference no. EKNZ UBE-15/49). Children were enrolled after their parents or caregivers gave written informed consent.

Infections with *Schistosoma* spp. were treated with praziquantel (40 mg/kg), soil-transmitted helminths with albendazole (200 or 400 mg depending on children’s age), and *Strongyloides stercoralis* with ivermectin (3 mg), immediately after diagnosis [21]. Additionally, children with a history of TB exposure without active disease were started on isoniazid preventive therapy (20 mg/kg) [22]. Children with anemia (hemoglobin <11 g/dl) were given iron or folic acid supplements, as clinically appropriate. In addition, dietary counseling was provided to parents and caregivers of all children with impaired nutritional status. Human immunodeficiency virus (HIV)-positive children were referred to a care and treatment center for further management, in line with Tanzanian guidelines.

### Study setting

The study was carried out in the Temeke district, Dar es Salaam, Tanzania [7] between October 2015 and September 2016. The district has routine TB contact tracing in place supported by TB patients who successfully completed treatment. Mass deworming in the district is coordinated by the neglected tropical disease control coordinator. Although the local water authority supplies piped water to the district, due to the high demand, residents also use ground water sources from boreholes for household chores which is vulnerable to pollution from pit latrines. This borehole water is used by most of the residents in the district [23].

### Study design

The current manuscript used the baseline data of a case-control study pertaining to the epidemiology of TB and helminth coinfections among children exposed and not exposed to TB. Preschool-aged children were recruited from households with an adult TB case (sputum smear-positive for acid-fast bacilli) and from TB-free neighboring households (to serve as controls), based on previously described standard operating procedures [24]. In the present cross-sectional study embedded within the aforementioned case-control study, we assessed the prevalence of helminth infections and determined associations with household characteristics, child development and cognition, and hematological factors in the surveyed children.

### Study population and sample size

We aimed for a sample size of 308 children, aged 6–59 months, with 154 TB-exposed and 154 TB-unexposed preschool-aged children, and with one child recruited per household. This sample size would allow estimating local helminth prevalence with a precision of 5% and at an error probability of 5% if the helminth prevalence were of the order of 30%.

### Study procedures

Children were seen by trained study clinicians who collected sociodemographic and socioeconomic information and obtained their medical history, including prior illnesses and use of medication. Clinicians assessed children for TB signs and symptoms [22]. A TB-exposure score chart from South Africa was employed to assess TB exposure [25]. The TB score was then categorized into (i) not likely to have TB infection (score of 1–6), or (ii) presumptively TB infected (score of ≥7). In addition, all children had a chest X-ray done. Trained study nurses recorded anthropometric measurements (height and weight), collected samples (blood, urine, stool, adhesive tape slide, and induced sputum), and performed development and cognitive assessments (gross motor, fine motor, language, and social components).

On the day of enrollment, parents or caregivers were given two empty containers labeled with the participant’s unique identification number and invited to submit one fresh morning stool sample and one urine sample of their child the following day. The samples were transferred to a nearby laboratory within 3 hours of collection. Due to limited financial and human resources, only a single stool and urine sample could be collected. Additionally, each participant was provided with a plastic pocket that contained an adhesive tape (50 x 20 mm) and a pre-labeled glass slide and asked to submit the slide with the anal adhesive tape for *Enterobius vermicularis* examination as described elsewhere [26]. We collected venous blood samples for full blood cell (FBC) counts and for malaria and HIV screening, along with induced sputum samples for microbiological investigation. All samples were received at Temeke clinic, transferred to a laboratory in appropriate temperature-controlled cooler boxes, and processed within 5 hours of receipt.

### Cognitive assessment

A validated Malawi Development Assessment Tool (MDAT) that was translated into Kiswahili was used to assess children’s development and cognition [27]. A medical doctor with expert training in pediatrics [28] trained the study nurses before commencing the study. Monthly refresher trainings were conducted on site for the duration of the study. Each child was assessed for 40 min. Parents or caregivers of acutely ill children were advised to return within a week of the child’s recovery for assessment [28].

### Laboratory procedures

#### Helminth investigations

A single stool sample was obtained from each child, subjected to triplicate Kato-Katz thick smears, and examined under a microscope by trained laboratory technicians for species-specific diagnosis of helminth infection. Triplicate Kato-Katz thick smear slides and the FLOTAC methods were employed for the diagnosis of *Ascaris lumbricoides*, hookworm, *Hymenolepis diminuta*, *Schistosoma mansoni*, and *Trichuris trichiura* while the Baermann technique was used to detect larvae of *Strongyloides stercoralis* [29]. The adhesive tapes were examined under a microscope for *E*. *vermicularis* eggs [26]. To screen for *S*. *haematobium* eggs, urine samples underwent urine filtration in duplicates using a hydrophilic polycarbonate membrane filter with a pore size of 20 μm (Sterlitech; Kent, United States of America) and subsequent examination of the filters for *S*. *haematobium* eggs. Microhematuria was examined by reagent strips (Hemastix; Siemens Healthcare Diagnostics, Eschborn, Germany). Urine samples were additionally tested for *Schistosoma* spp. antigens using a point-of-care circulating cathodic antigen (POC-CCA) cassette test (Rapid Medical Diagnostics; Pretoria, South Africa) which has been primarily validated for *S*. *mansoni*, but cross-reactivity has been reported [20, 30]. Using a visual aid tool and based on a semi-quantitative score, the POC-CCA results were interpreted as negative, trace, 1+, 2+, or 3+. All slides with adhesive tapes, Kato-Katz thick smears, and urine filters were stored in boxes, and 10% of the slides were re-examined for quality control purposes by experienced laboratory technicians within 6 months [29]. All helminth investigations were conducted at the Bagamoyo Research and Training Centre. The standard operating procedures have been described in detail elsewhere [31].

#### Microbiological investigations

Xpert MTB/RIF (Cepheid; Sunnyvale, CA, United States of America) was performed on induced sputum samples at the Temeke district hospital laboratory to aid in the diagnosis of TB. The laboratory is continuously monitored for quality by the Central Tuberculosis Reference Laboratory (Dar es Salaam, Tanzania).

#### Blood testing

Blood samples were screened for malaria with a rapid diagnostic test (Access Bio; Somerset, NJ, United States of America), and for HIV infection using Alere Determine HIV-1/2 (Alere; Waltham, MA, United States of America) if the child’s age was ≥18 months or RNA polymerase chain reaction if <18 months. The FBC were done with an MS4 Vet hematology analyzer (Diamond Diagnostics; Massachusetts, United States of America) to determine hematological indices such as hemoglobin, mean corpuscular volume (MCV), mean corpuscular hemoglobin (MCH), and red blood cell distribution width (RCDW).

### Data collection and definitions

Data were recorded into tablet computers, using open data kit (ODK; http://opendatakit.org/) and “odk_planner”, a data management tool. Laboratory results were entered into ODK from paper forms.

A helminth infection was defined as positive when eggs or larvae of the following species were microscopically identified: *A*. *lumbricoides*, *E*. *vermicularis*, hookworm, *H*. *diminuta*, *S*. *haematobium*, *S*. *mansoni*, *S*. *stercoralis*, or *T*. *trichiura*. Subsequently, helminth infections were grouped into (i) schistosomiasis, defined as infection with either *S*. *mansoni* or *S*. *haematobium* (based on stool microscopy, using Kato-Katz thick smears, urine filtration and/or positive POC-CCA urine cassette test results) and (ii) other helminthiases, including infections with any of the other helminths (*A*. *lumbricoides*, *H*. *diminuta*, hookworm, *T*. *trichiura*, *E*. *vermicularis*, and *S*. *stercoralis*). A POC-CCA test was regarded as positive if the band revealed 1+, 2+, or 3+. In sensitivity analyses, POC-CCA definition included also trace-positive results.

In the absence of any signs or symptoms suggestive of TB and/or as ascertained by Xpert MTB/RIF, a child was considered presumptively TB infected if the TB exposure score was ≥7 and unlikely to have a TB infection if the score was 1–6 [25]. Anemia was defined as hemoglobin <11.0 g/dl, as per WHO recommendations [32]. Anthropometric z-scores were calculated using the 2006 WHO Growth Standards in Stata version 13.1 (Stata Corporation; College Station, TX, United States of America) using the ‘zscore06’ command [33].

### Statistical analysis

Absolute frequencies and proportions were used to describe children, parents/caretakers, and household characteristics overall and stratified by the two groups of helminthiases. A measure of socioeconomic status was derived from a factor analysis of household asset variables and defined as low or high for score values below and above the median, respectively. Clinical outcomes included anemia, cognitive score and anthropometric measures (weight and height). We performed mixed logistic regression analyses with random intercepts at the level of matched pairs to identify risk factors for helminth infection, considering schistosomiasis and other helminthiases. We constructed multivariable core models comprising age, sex, type of toilet, hygiene behavior, and parent education variables based on clinical relevance and added other variables as appropriate, one by one. We also performed a sensitivity analysis to identify risk factors for *Schistosoma* spp. infection using the core model as above and considering trace results in the POC-CCA urine cassette test as positive. We used box-plots to compare the four MDAT components in children with and without helminth infections and calculated the overall median and interquartile range (IQR) of the total MDAT score and across relevant subsamples. We dichotomized the four components of the MDAT score at their median and ran mixed logistic regressions to compare scores between helminth-infected and uninfected children. We also compared hematological indices according to the presence of helminth infections using mixed linear regression models. All analyses were performed in Stata version 13.1 (Stata Corporation; College Station, United States of America).

## Results

### Study flow and baseline characteristics of children

**Fig 1:**
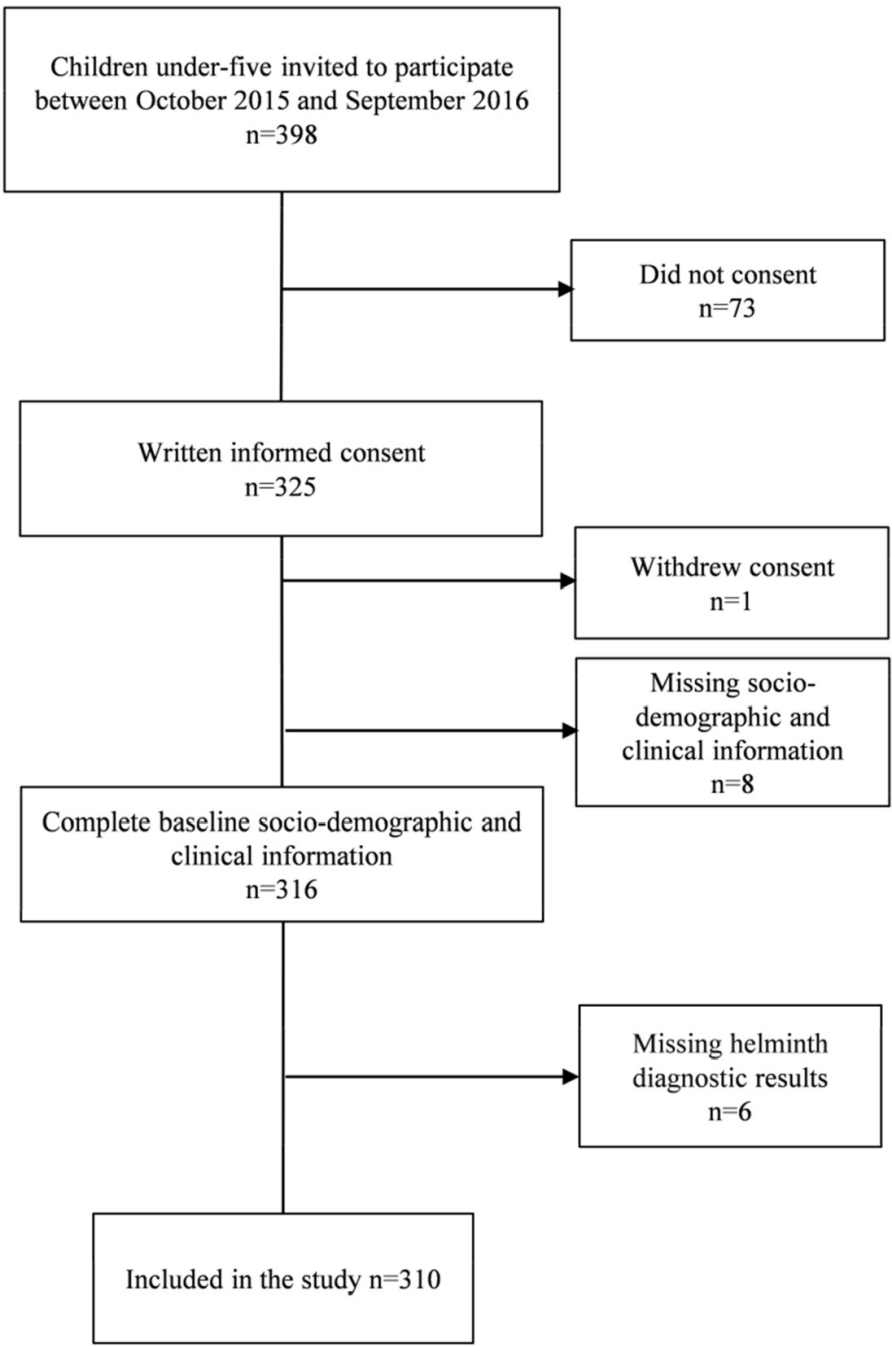
Flow chart of the 310 participants included in the study.

We invited 398 parents and caregivers with children aged 6–59 months to participate. Parents/caregivers of 325 children consented and their children were enrolled. Of those, 310 completed the study procedures. Eight children did not provide their sociodemographic and clinical information, six did not submit stool and urine samples for helminth diagnosis, and one parent withdrew consent (Fig 1).

Of the 310 participating children, 160 (52%) were girls and the median age was 26 months (IQR: 17–42 months, range 6–58 months). The median height-for-age Z-score (HAZ) was -1.14 (95% confidence interval (CI): -1.91 to -0.20) (Table 1). A total of 189 (61%) children were exposed to smear-positive adult pulmonary TB patients and four (1.3%) were HIV-positive. Twenty-nine (9.4%) mothers reportedly tested HIV-positive during pregnancy. Fourteen (4.5%) children had a positive malaria rapid diagnostic test, six (1.9%) reportedly received anthelmintics within 3 months prior to enrollment in the study. Parents/caretakers of 23 (7.4%) children reported having moved from other regions to Dar es Salaam after their children were born.

**Table 1:** Baseline sociodemographic, socioeconomic, and clinical characteristics of 310 preschool-aged children in a study conducted between October 2015 and September 2016, and their parents/caregivers in the Temeke district, Dar es Salaam, Tanzania.

Characteristic	All	Any helminth species ^1^		*Schistosoma* spp. ^2^	
n (%)	(n = 310)				
		Infected (n = 74)	Not infected (n = 236)	Infected (n = 49)	Not infected (n = 261)
**Child characteristics**					
**Age (months), median (IQR)**	26 (17–42)	23 (17–36)	28 (17–43)	23 (18–38)	27 (16–42)
**Age groups (months)**					
6–12	52 (17)	11 (15)	41 (17)	8 (16)	44 (17)
13–24	92 (30)	29 (39)	63 (27)	18 (36)	74 (28)
25–36	71 (23)	16 (22)	55 (23)	10 (21)	61 (24)
37–48	57 (18)	10 (14)	47 (20)	7 (14)	50 (19)
49–59	38 (12)	8 (11)	30 (13)	6 (13)	32 (12)
**Sex**					
Female	160 (52)	37 (50)	123 (52)	24 (49)	136 (52)
Male	150 (48)	37 (50)	113 (48)	25 (51)	125 (48)
**Delivered by**					
Caesarean section	40 (13)	13 (17)	27 (11)	10 (20)	30 (12)
SVD	253 (20)	56 (76)	197 (84)	38 (76)	215 (82)
Unknown	17 (5)	5 (7)	12 (5)	1 (2)	16 (6)
**Born at gestation age (weeks)**					
Pre-term <37	9 (3)	2 (2)	7 (3)	1 (2)	8 (3)
Term ≥37	284 (92)	67 (91)	217 (90)	45 (96)	239 (91)
Unknown	17 (5)	5 (7)	12 (5)	1 (2)	61 (6)
**Birth weight (kg)**					
Low <2.5	28 (9)	5 (7)	24 (10)	4 (8)	25 (10)
Normal ≥2.5	265 (86)	64 (86)	200 (85)	44 (09)	220 (84)
Unknown	17 (5)	5 (7)	12 (5)	1 (2)	16 (6)
**Immunization status**					
BCG, with scar	306 (99), 260 (85)	71 (96)	235 (99)	42 (86)	218 (84)
Measles	263 (85)	68 (92)	195 (83)	46 (94)	217 (83)
**HIV status**					
Positive	4 (1.3)	2 (3)	2 (1)	0	4 (2%)
Negative	306 (98.7)	72 (97)	234 (99)	49 (100)	257 (98)
**Hemoglobin level (g/dl)**					
Anemic <11.0	203 (65)	56 (76)	147 (62)	35 (71)	168 (64)
Not anemic ≥11.0	104 (34)	17 (23)	87 (37)	13 (27)	91 (35)
Missing	3 (1)	1 (1)	2 (1)	1 (2)	2 (1)
**Malaria rapid diagnostic test**					
Positive	14 (5)	3 (4)	11 (5)	3 (6)	11 (4)
Negative	296 (95)	71 (96)	225 (95)	46 (94)	250 (96)
**TB exposure history**					
Exposed	189 (61)	46 (62)	143 (61)	33 (67)	156 (60)
Unexposed	121 (39)	28 (38)	93 (39)	16 (33)	105 (40)
**TB exposure score**					
Likely not infected	197 (64)	50 (68)	147 (62)	33 (67)	164 (63)
Likely infected	113 (36)	24 (32)	89 (38)	16 (33)	97 (37)
**Deworming status (past 3 months)**					
Not dewormed	304 (98)	72 (97)	232 (98)	48 (98)	256 (98)
Dewormed	6 (2)	2 (3)	4 (2)	1 (2)	5 (2)
**HAZ-scores**					
Median (IQR)	-1.14 (-1.91 to -0.2)	-1.16 (-1.72 to -0.07)	-1.12 (-1.94 to -0.33)	-1.17 (-1.58 to -0.13)	-1.09 (-1.98 to -0.31)
**WAZ-score**					
Median (IQR)	-1.14 (-2.07 to -0.35)	-1.3 (-2.22 to -0.28)	-1.12 (-1.99 to -0.35)	-1.34 (-2.36 to -0.69)	-1.10 (-2.05 to -0.33)
**WHZ-score**					
Median (IQR)	-0.94 (-2.02 to -0.13)	-1.16 (-2.02 to -0.17)	-0.79 (-1.86 to -0.13)	-1.48 (-2.07 to -0.10)	-0.75 (-1.83–15.0)
**Household characteristics**					
**Number of people**					
<6	190 (61)	43 (58)	147 (62)	29 (59)	161 (62)
≥6	120 (39)	31 (42)	89 (38)	20 (41)	100 (38)
**Household income per month (US$)**					
<100	108 (35)	28 (38)	80 (34)	15 (31)	93 (36)
≥100	202 (65)	46 (62)	156 (66)	34 (69)	168 (64)
**Water source for household chores**					
Bore well	90 (29)	15 (20)	27 (11)	14 (29)	28 (11)
Tap	153 (49)	43 (58)	158 (67)	27 (55)	174 (67)
Unknown	67 (22)	16 (22)	51 (22)	8 (16)	59 (22)
**Type of household toilet**					
Septic tank	93 (30)	28 (38)	65 (28)	21 (43)	72 (28)
Pit latrine	217 (70)	46 (62)	171 (72)	28 (57)	189 (72)
**Hygienic practices**					
Poor	36 (12)	12 (16)	24 (10)	7 (14)	29 (11)
Good	274 (88)	62 (84)	212 (90)	42 (86)	232 (89)
**SES**					
Low	159 (50)	40 (54)	119 (50)	28 (57)	131 (50)
High	151 (50)	34 (46)	127 (50)	21 (43)	130 (50)
**Parent/caregiver characteristics**					
**Mothers prior pregnancies**					
Unknown	17 (5)	5 (7)	12 (5)	1 (2)	16 (6)
0	88 (30)	19 (26)	69 (29)	9 (19)	79 (30)
1–2	142 (48)	39 (53)	103 (44)	30 (64)	112 (43)
≥3	63 (17)	11 (15)	52 (22)	7 (15)	56 (21)
**Mothers HIV status during pregnancy**					
Unknown	24 (8)	6 (8)	18 (7)	1 (2)	23 (9)
Positive	29 (9)	4 (5)	25 (11)	3 (6)	26 (10)
Negative	257 (83)	64 (86)	193 (82)	45 (92)	212 (81)
**Mothers marital status**					
Single	76 (25)	19 (25)	57 (24)	14 (29)	62 (24)
Married	217 (70)	50 (68)	167 (71)	34 (69)	183 (70)
Unknown	17 (5)	5 (7)	12 (5)	1 (2)	16 (6)
**Parent education level**					
No or primary education	244 (79)	63 (85)	181 (77)	42 (86)	202 (77)
Secondary/higher education	66 (21)	11 (15)	55 (23)	7 (14)	59 (23)
**Parent occupation**					
Unemployed	196 (63)	49 (66)	147 (62)	31 (63)	165 (63)
Employed	114 (37)	25 (34)	89 (38)	18 (37)	96 (37)
**Family migration history since child birth**					
Migrated	23 (7)	8 (11)	15 (6)	3 (6)	20 (8)
Did not migrate	189 (61)	44 (59)	144 (61)	36 (73)	153 (58)
Unknown	98 (32)	22 (30)	77 (33)	10 (20)	88 (34)

HAZ, height for age, moderate to severe stunting (z-score≤-2); HIV, human immunodeficiency virus; TB exposure score based on Mandalakas et al. [25]; SVD, spontaneous vaginal delivery; WAZ, weight for age, moderate to severe underweight (z-score≤-2); WHZ, weight for height, moderate to severe wasting (z-score≤-2); US**$**, United States dollars (1 US**$** = 2,190 Tanzanian Shillings); SES, socioeconomic status (low = below median of the principal asset score, high = above the median of the principal asset score)

^**1**^ Any helminth infection defined as positive when eggs or larvae of the following species were microscopically identified: *A*. *lumbricoides*, *E*. *vermicularis*, hookworm, *H*. *diminuta*, *S*. *haematobium*, *S*. *mansoni*, *S*. *stercoralis*, or *T*. *trichiura*; or a positive POC-CCA urine cassette test result indicating *Schistosoma* spp. infection (test result 1+, 2+, or 3+)

^**2**^ *Schistosoma* spp. includes *S*. *mansoni* and *S*. *haematobium*

### Prevalence of helminth infections

The overall prevalence of *Schistosoma* spp. infection was 15.8% (95% CI 12.1–20.3%). *Schistosoma* spp. infection as determined by POC-CCA, was found in 47 children (15.2%; 95% CI 11.6–19.6%), *S*. *haematobium* eggs were only found in the urine of three individuals (1.0%) (Table 2), and no *S*. *mansoni* eggs were found in any of the Kato-Katz thick smears or FLOTAC examinations. There was no difference in the distribution of children with *Schistosoma* spp. infection in young (6–24 months) and older (25–59 months) age groups (53% *vs*. 47%, p = 0.3) or between boys and girls (51% *vs*. 49%, p = 0.7). There was also no significant difference between TB-exposed and unexposed children (67% *vs*. 60%, p = 0.3), as shown in Table 1. The prevalence of *Schistosoma* spp. infection (as determined by POC-CCA) increased to 31.0% (95% CI 26.3–36.7%) when considering trace results as positive.

**Table 2:** Frequency distribution of helminth species among preschool-aged children in Dar es Salaam, Tanzania in a study conducted between October 2015 and September 2016.

Helminth infection	All	≤24 months		>24 months	
		Male	Female	Male	Female
	n (%)	n (%)	n (%)	n (%)	n (%)
**Total**	310 (100)	72 (100)	72 (100)	78 (100)	88 (100)
**Any helminth infection** ^**1**^	74 (23.9)	25 (34.7)	15 (20.8)	12 (15.4)	22 (25.0)
**Schistosomiasis**					
***Schistosoma* spp. (POC-CCA)** ^**2**^					
Any positive result (trace and positive)	97 (31.3)	27 (37.5)	21 (29.2)	20 (25.6)	29 (33.0)
Trace	50 (16.1)	11 (15.3)	12 (16.7)	11 (14.1)	16 (18.2)
Positive	47 (15.2)	16 (22.2)	9 (12.5)	9 (11.5)	13 (14.3)
1+	34 (11.0)	8 (11.1)	9 (12.5)	6 (7.7)	11 (12.5)
2+	12 (3.9)	8 (11.1)	0 (0.0)	2 (2.6)	2 (2.3)
3+	1 (0.3)	0 (0.0)	0 (0.0)	1 (1.3)	0 (0.0)
***Schistosoma haematobium*** ^**3**^					
Positive	3 (0.97)	1 (1.5)	1 (1.5)	0 (0.0)	1 (1.1)
**Other helminth infection** ^**4**^					
Any of the other helminth species	28 (9.0)	10 (13.9)	5 (6.9)	4 (5.1)	9 (10.2)
*Strongyloides stercoralis*	16 (5.2)	6 (8.3)	3 (4.2)	3 (3.9)	4 (4.6)
*Enterobius vermicularis*	6 (1.9)	1 (1.4)	1 (1.4)	1 (1.3)	3 (3.4)
Hookworm	6 (1.9)	3 (4.2)	2 (2.8)	0 (0.0)	1 (1.1)
*Ascaris lumbricoides*	1 (0.3)	0 (0.0)	0 (0.0)	0 (0.0)	1 (1.1)
*Hymenolepis diminuta*	1 (0.3)	0 (0.0)	0 (0.0)	0 (0.0)	1 (1.1)

^**1**^ Any helminth was defined as positive when eggs or larvae of the following species were microscopically identified: *A*. *lumbricoides*, *E*. *vermicularis*, hookworm, *H*. *diminuta*, *S*. *haematobium*, *S*. *mansoni*, *S*. *stercoralis*, and *T*. *trichiura*

^**2**^ Point-of-care circulating cathodic antigen urine cassette test for detection of *Schistosoma* spp. infection (POC-CCA test result 1+, 2+, or 3+).

^**3**^ Based on urine filtration (egg-positive urine filtration)

^**4**^ Other helminth species (based on stool or adhesive tape microscopy): *A*. *lumbricoides*, *E*. *vermicularis*, hookworm, *H*. *diminuta*, and *S*. *stercoralis*

Five participants had dual species and one participant a triple species helminth infection

The prevalence of other helminth species infections, excluding *Schistosoma* spp., was 9.0% (95% CI 6.3–12.8%). The most frequently detected helminth species was *S*. *stercoralis* (16 children; 5.2%), followed by *E*. *vermicularis* (6; 1.9%), and hookworm (6; 1.9%). Infections with *A*. *lumbricoides* and *H*. *diminuta* were found in only one child each, and no *T*. *trichiura* infection was observed (Table 2). The difference in the distribution of helminth infections between TB-exposed and unexposed children was not statistically significant (62% *vs*. 54%, p = 0.4).

Five children (1.6%) had dual species helminth infections: two with *Schistosoma* spp.-*S*. *stercoralis*; and one each with *Schistosoma* spp.-*E*. *vermicularis*, *E*. *vermicularis*-hookworm, and *A*. *lumbricoides*-*H*. *diminuta*. One child had a triple species helminth infection with *Schistosoma* spp.-*E*. *vermicularis*-hookworm.

### Risk factors for helminth infections

*Schistosoma* spp. infection was significantly associated with having a septic tank toilet in the household (adjusted odds ratio (aOR) 2.04, 95% CI: 1.02–4.07, p = 0.042; Table 3). Higher education of parents/caregivers, tap water at home, and better hygiene practices showed no significant association with *Schistosoma* spp. infection. Additionally, *Schistosoma* spp. infection was similar in TB-exposed and unexposed children (aOR 1.34, 95% CI: 0.67–2.68, p = 0.4) (Table 3). In the sensitivity analysis that considered POC-CCA trace results as positive, none of the variables included in the core model, including having septic tank toilets, were associated with *Schistosoma* spp. infection (S1 Table). Furthermore, none of the risk factors were significantly associated with any of the other helminth infection, including having a septic tank toilet (aOR 0.92, 95% CI: 0.35–2.40, p = 0.9) (Table 3).

**Table 3:** Risk factors for *Schistosoma* and soil-transmitted helminth infections among preschool-aged children in Dar es Salaam, Tanzaniain a study conducted between October 2015 and September 2016.

Characteristics	All	*Schistosoma* spp.				Other helminths			
	n (%)	Crude		Adjusted		Crude		Adjusted	
		OR (95% CI)	p value	aOR (95% CI)	p value	OR (95% CI)	p value	aOR (95% CI)	p value
**Age groups (months)**			0.5		0.5		0.3		0.3
6–12	52 (17)	1.00		1.00		1.00		1.00	
13–24	92 (30)	1.33 (0.21–3.40)		1.31 (0.51–3.40)		2.36 (0.56–9.86)		2.38 (0.58–9.78)	
25–36	71 (23)	0.89 (0.31–2.51)		0.86 (0.30–2.44)		2.00 (0.45–8.92)		1.91 (0.44–8.36)	
37–48	57 (18)	0.76 (0.25–2.34)		0.76 (0.24–2.37)		0.80 (0.13–4.78)		0.78 (0.13–4.59)	
49–59	38 (12)	1.00 (0.31–3.32)		0.92 (0.27–3.11)		0.80 (0.11–5.83)		0.80 (0.11–5.72)	
**Sex**			0.7		0.8		0.7		0.8
Female	160 (52)	1.00		1.00		1.00		1.00	
Male	150 (48)	1.12 (0.59–2.11)		1.06 (0.55–2.02)		1.16 (0.47–2.85)		1.05 (0.44–2.51)	
**Individual deworming history**^**1**^			0.9		0.9		0.6		0.6
Not dewormed	304 (98)	1.00		1.00		1.00		1.00	
Dewormed	6 (2)	1.07 (0.11–10.12)		1.06 (0.11–10.1)		2.14 (0.17–26.84)		2.24 (0.18–27.20)	
**TB exposure**			0.3		0.4		0.5		0.6
Unexposed	121 (39)	1.00		1.00		1.00		1.00	
Exposed	189 (61)	1.43 (0.73–2.82)		1.34 (0.67–2.68)		0.72 (0.31–1.67)		0.74 (0.31–1.74)	
**Number of people in the household**			0.7		0.7		0.4		0.4
<6	190 (61)	1.00		1.00		1.00		1.00	
≥6	120 (39)	1.13 (0.59–2.16)		1.13 (0.58–2.20)		1.47 (0.61–3.53)		1.49 (0.62–3.54)	
**Water source for household chores**			0.2		0.3		0.3		0.3
Bore well	90 (29)	1.00		1.00		1.00		1.00	
Tap	153 (49)	0.43 (0.21–0.88)		0.43 (0.20–0.94)		4.02 (1.04–15.5)		4.12 (1.05–16.3)	
Unknown	67 (22)	0.41 (0.16–1.02)		0.41 (0.16–1.08)		4.22 (0.96–18.5)		4.90 (1.07–22.3)	
**Type of toilet**			0.04		0.04		0.9		0.9
Pit latrine	217 (70)	1.00		1.00		1.00		1.00	
Septic tank	93 (30)	2.03 (1.03–4.00)		2.04 (1.02–4.07)		0.98 (0.37–2.57)		0.92 (0.35–2.40)	
**Hygienic practices**^**2**^			0.5		0.8		0.3		0.3
Poor	36 (12)	1.00		1.00		1.00		1.00	
Better	274 (88)	0.74 (0.29–1.87)		0.87 (0.34–2.25)		0.55 (0.17–1.82)		0.54 (0.16–1.78)	
**Household income per month (US$)**^**3**^			0.5		0.3		0.3		0.4
<100	108 (35)	1.00		1.00		1.00		1.00	
≥100	202 (65)	1.25 (0.63–2.45)		1.56 (0.78–3.13)		0.61 (0.26–1.44)		0.71 (0.29–1.73)	
**Parent education level**			0.2		0.2		0.4		0.5
No or primary education	244 (79)	1.00		1.00		1.00		1.00	
Secondary/higher education	66 (21)	0.57 (0.24–1.36)		0.53 (0.22–1.28)		0.60 (0.18–1.97)		0.63 (0.19–2.09)	
**Parent occupation**			0.9		0.7		0.4		0.5
Housewife/unemployed	196(63)	1.00		1.00		1.00		1.00	
Employed	114(37)	1.01 (0.52–1.96)		0.85 (0.42–1.76)		0.65 (0.25–1.66)		0.70 (0.26–1.92)	
**Family migration history since child birth**			0.08		0.1		0.07		0.1
Migrated	23 (7)	1.00		1.00		1.00		1.00	
Did not migrate	189 (61)	1.58 (0.43–5.84)		1.55 (0.41–5.80)		4.74 (1.28–17.63)		5.30 (1.43–9.74)	
Unknown	98(32)	0.74 (0.18–3.09)		0.74 (0.18–3.10)		2.24 (0.89–5.63)		2.04 (0.80–5.18)	

Schistosomiasis includes *S*. *mansoni* and *S*. *haematobium* (positive POC-CCA urine cassette test results 1+, 2+, or 3+ and egg-positive urine filtration); other helminth species (based on stool microscopy) include *A*. *lumbricoides*, *E*. *vermicularis*, hookworm, *H*. *diminuta*, and *S*. *stercoralis*

^1^ Past 3 months

^2^ Hygiene practice: parent/caregiver always wash fruits or vegetables before giving to children

^3^ US**$**, United States dollars (1 US**$** = 2,190 Tanzanian shillings)

Multivariable mixed logistic regression model with random intercepts at the level of matched pairs, containing the respective variable along with age, sex, and type of toilet

### Association of helminth infections with development and cognitive scores

The overall median MDAT score in the study population was 3.30 (IQR 2.78–3.49). There was no significant difference in the overall median cognitive score in helminth-infected and uninfected children (3.20 [95% CI 2.74–3.44] *vs*. 3.33 [95% CI 2.80–3.50], p = 0.2 (S2 Table). There was also no effect of *Schistosoma* spp. infection on the overall median cognitive score among the two groups (3.17 [95% CI 2.78–3.44] *vs*. 3.32 [95% CI 2.78–3.50], p = 0.2).

**Fig 2:**
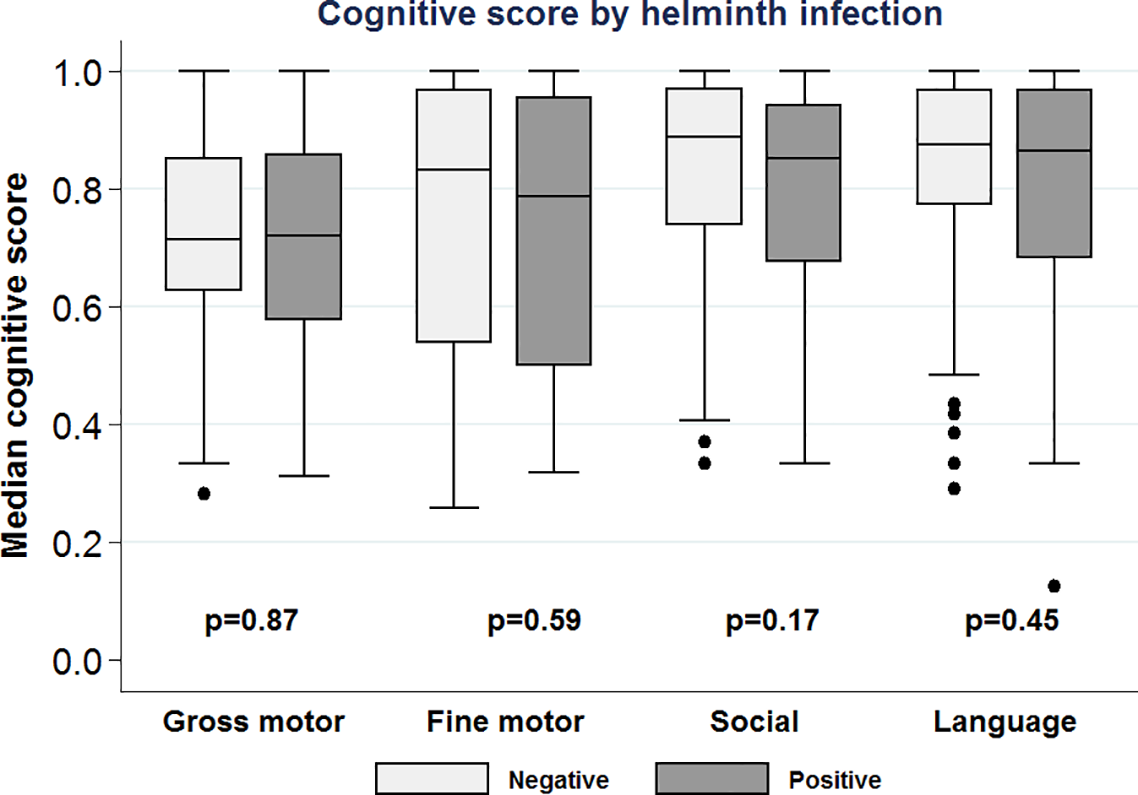
Box-plots comparing development and cognitive function among children with and without helminth infection.

The median gross motor score tended to be higher among preschool-aged children with a helminth infection compared to their uninfected peers. The median fine motor (0.79 *vs*. 0.83), social (0.85 *vs*. 0.89), and language scores (0.86 *vs*. 0.88) tended to be lower among helminth-infected compared to helminth-uninfected children (Fig 2), but none of the differences achieved statistical significance.

### Association of helminth infection with hematological parameters

Almost two-third of the children (203; 65%) were anemic; nine (4.4%) of those with anemia had a positive rapid malaria diagnostic test result. Moderate anemia (hemoglobin level 7.0–9.9 g/dl) was most prevalent (49%), while mild anemia (hemoglobin 10.0–10.9 g/dl) was found in 44%, and severe anemia (hemoglobin <7 g/dl) was found in 14 of the anemic children (6.9%). Five (6%) children with mild anemia, three (3%) with moderate anemia, and one (7%) with severe anemia had malaria.

Anemia was diagnosed in 56 (77%) participants with helminth infections, including all six with hookworm, all three with *S*. *haematobium*, the one with *A*. *lumbricoides*, and the one with *H*. *diminuta*. With regard to *Schistosoma* spp. infection (as determined by POC-CCA), 33 out of 46 infected had anemia (72%) and 12 of the 16 *S*. *stercoralis*-infected children were anemic (75%). Two children with anemia had both helminth infections and malaria.

**Fig 3:**
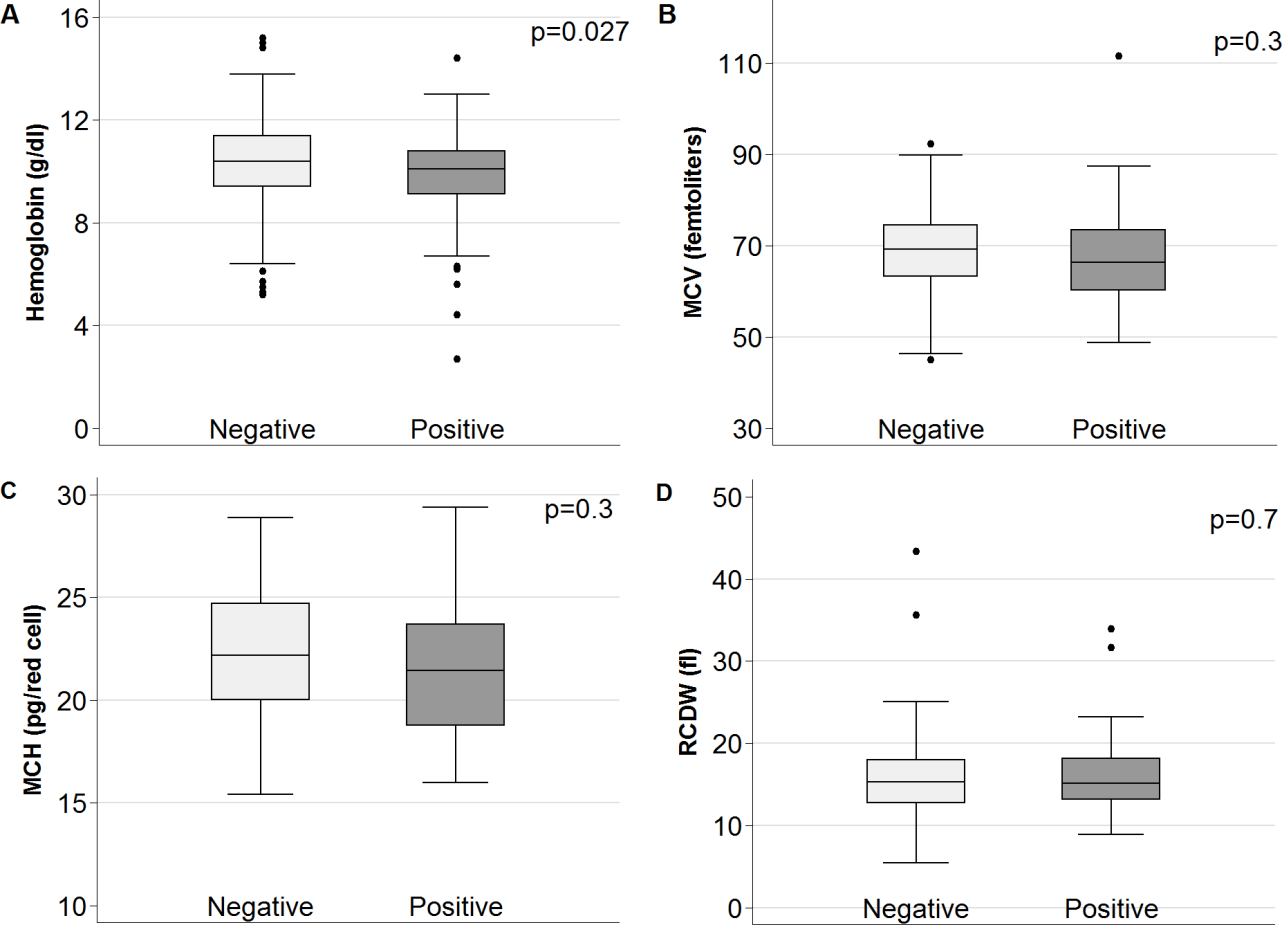
Box plots showing distribution of hemoglobin and red blood cell indices among children with (n = 73) and without helminth infection (n = 234). A) Distribution of hemoglobin by helminth infection; B) distribution of mean corpuscular volume by helminth infection; C) distribution of mean corpuscular hemoglobin by helminth infection; and D) distribution of red blood cell distribution width by helminth infection.

When comparing hemoglobin and hematological parameters with helminth infection, the median hemoglobin value was significantly lower in helminth-infected children compared with their uninfected peers (10.1 g/dl [IQR 9.1–10.8 g/dl] *vs*. 10.4 g/dl [IQR 9.4–11.4 g/dl], p = 0.027) (Fig 3). This difference remained significant even when excluding malaria cases (10.4 g/dl [IQR 9.6–11.4 g/dl] *vs*. 10.1 g/dl [IQR 9.0–10.8], p = 0.014). All other hematological parameters (MCV, MCH, and RCDW) were equally distributed between helminth-infected and uninfected children.

## Discussion

We present findings on the prevalence, clinical relevance, and risk factors associated with helminth infection among preschool-aged children in a poorly planned and under-resourced district in the coastal region of Dar es Salaam, Tanzania. We found that the prevalence of *Schistosoma* spp. was high (16.0%) among children under the age of 5 years, but the prevalence of other helminth infections was relatively low. We found no positive associations between helminth infections and commonly reported risk factors or development/cognitive scores. Anemia was a common clinical presentation and more frequent among children infected with helminths than their non-infected counterparts.

To our knowledge, this is the first study to report such a high prevalence of *Schistosoma* spp., as determined by the POC-CCA urine cassette test among preschool-aged children in the coastal urban area of Dar es Salaam. The POC-CCA is considered a highly sensitive rapid diagnostic test and was primarily developed for the detection of *S*. *mansoni* [20]. In Tanzania, the POC-CCA has previously been used among preschool-aged children to detect *S*. *mansoni*, reporting a high prevalence of up to 50% in well-known high-risk *S*. *mansoni* areas around Lake Victoria (North-Western part of Tanzania), where the natural open freshwater serves as a habitat for the intermediate host snails [34, 35]. However, a recent systematic review highlighted a low specificity of the POC-CCA test assay in detecting *S*. *mansoni* (as compared with stool microscopy) and/or the possibility of cross-reactivity of the assay with *S*. *haematobium* [30]. In our study, the positive POC-CCA results were not confirmed by stool microscopy, since the commonly used Kato-Katz method failed to identify any *S*. *mansoni* eggs in our study population. Furthermore, the urine filtration only revealed a very low prevalence of *S*. *haematobium* (1.0%). Similarly, in a recent investigation in Dar es Salaam that used Kato-Katz and urine filtration but not the POC-CCA, the prevalence of *S*. *haematobium* among school-aged children was reported to be 1.2%, while no *S*. *mansoni* was reported [36]. Likely, the conventional stool and urine examination underestimate the true prevalence due to their low sensitivity to detect light intensity infection as they might occur in young children. However, an overestimation of *Schistosoma* spp. prevalence by a potential cross-reactivity of the POC-CCA with other conditions can also not be fully ruled out [30].

Urban schistosomiasis caused by *S*. *mansoni* has been reported elsewhere, including Brazil [37], Côte d’Ivoire [38] and Tanzania [39], but most of these studies did not include preschool-aged children. However, intense transmission of *S*. *mansoni* has never been formally demonstrated in urban regions of Tanzania such as Dar es Salaam [40, 41]. Dar es Salaam is a coastal city along the Indian Ocean and it was known to have a high prevalence and transmission of *S*. *haematobium* since the 1980s [34, 40]. Our study showed that the prevalence of *S*. *haematobium* and *S*. *mansoni* infection as determined by egg counts in urine and stool is low, while the POC-CCA suggests that infections due to *Schistosoma* spp. have a considerably higher prevalence. Further studies using highly sensitive and specific tests for schistosomiasis diagnosis in coastal Tanzania involving different age and population groups should be conducted to establish the species- and age-specific prevalence as the global focus is shifting toward disease elimination.

Overall, the prevalence of other helminth infections was found to be lower than that reported in other under-resourced settings [16, 42]. Ten years ago, a study in two district hospitals in Dar es Salaam reported a soil-transmitted helminth prevalence (including hookworm, *A*. *lumbricoides*, and *T*. *trichiura*) of 33% among children below the age of 5 years [43]. The lower rates noted in our study may be due to an improved socioeconomic status among the general population and/or to successful biannual preventive chemotherapy campaigns, initiated in 2004, that include administering mebendazole and vitamin A supplementation to preschool-aged children [44].

We did not find any association between helminth infections and commonly reported risk factors such as age, hygiene, low socioeconomic status, and history of migration. This is in contrast to other studies, which identified age, poor hygiene, and low socioeconomic status as risk factors for helminth infection in children [16, 17, 35, 45]. The lack of association with risk factors might be in part due to our sampling strategy, which was primarily powered to detect the prevalence of helminth infection among our study population, rather than association with risk factors. Although we identified having toilets with septic tanks as a risk factor for *Schistosoma* spp. infection, this association lacked statistical significance after including POC-CCA trace results. We did not find evidence of an association between helminth infection and TB exposure. To our knowledge, no study has yet specifically investigated schistosomiasis and TB in preschool-aged children. However, a study in Kenya reported increased odds of hookworm infection among school-aged children with latent TB infection compared to unexposed controls [6]. It will be important to further elucidate the impact of helminth co-infections in early childhood on developing TB.

We documented a high prevalence of anemia among preschool-aged children that was associated with helminth infection. Similar findings have been reported in studies from Ethiopia and Nigeria, where children who were infected with two or more helminth species were at higher risk of having anemia [46]. High prevalence of anemia among preschool-aged children might also be caused by poor diets, low socioeconomic status of parents or caregivers, as indicated by the high rate of unemployment [23, 47]. Other assessed hematological parameters were not associated with helminth infection, possibly due to low prevalence and intensity of helminth infection as well as to the good nutritional status among children evidenced by HAZ and WAZ in our study [48]. Previous research showed that heavy helminth infection impairs development and cognition [10,49]. In our study, helminth infection was not associated with reduced development and cognition. However, such differences may be seen only over longer time frames during detailed follow-up surveys.

Our study has strengths and limitations that warrant further consideration. We systematically screened for helminthiases and other diseases, such as malaria, HIV, and active TB, using a suite of standardized and quality-controlled diagnostic tests [25, 34, 50]. These infectious diseases all contribute to high morbidity and mortality among children <5 years [11]. The main limitations of our study include sampling households based on TB exposure (given that the overall study aim was to explore interactions of TB and helminth co-infections), and restricting the study area to an urban setting. However, poorly planned urban settings have the highest population growth in sub-Saharan Africa with considerable disease burdens of major infectious and non-communicable diseases [51].

In conclusion, our study showed high prevalence of *Schistosoma* spp. infection as determined by the POC-CCA urine cassette test, among preschool-aged children, even in a highly urbanized setting in East Africa, an observation that has not been previously reported. It must be noted though that this result was achieved with a highly sensitive diagnostic assay, namely, the POC-CCA urine cassette test. Cross-reactivity with other conditions cannot be ruled out. Helminth infections were associated with anemia, but not with growth development and development of cognitive functions among our group of young children. However, the fact that helminth infection was not shown to affect children’s development and cognition does not mean they will not be affected later in life. With the WHO’s ambitious goal of reaching 100% coverage of preventive chemotherapy targeting major helminthiases, our findings call for urgent planning and implementation of specific interventions to prevent further morbidity, and to improve health, care, and wellbeing of these young children. Deworming likely reduces the prevalence of anemia, improves children’s development and cognition, and prevents complications later in life [46, 52]. Future research to confirm our findings using newly developed and highly sensitive and specific test assays, to identify and map *Schistosoma* spp. infection hotspots and its intermediate host snails in Dar es Salaam are needed to design targeted interventions for effectively controlling morbidity due to schistosomiasis and shift toward interruption of transmission.

## Supporting information

S1 Checklist STROBE checklist. (DOCX)

S1 Table Additional analysis: Risk factors for *S*. *mansoni* infection (defined trace results as positive based on point-of-care circulating cathodic antigen (POC-CCA) urine cassette test) among 310 under-five children in Temeke district, Dar es Salaam, Tanzania. (DOCX)

S2 Table Comparison of cognitive score among helminth-infected and non-infected preschool-aged children in Dar es Salaam, Tanzania. (DOCX)
