# Supplementary material for: Schistosoma, other helminth infections, and associated risk factors in preschool-aged children in urban Tanzania
Source: PLoS Negl Trop Dis. 2017 Nov 6;11(11):e0006017. doi: 10.1371/journal.pntd.0006017 (PMC5697890; doi:10.1371/journal.pntd.0006017)
Supplement: S1 Checklist — (DOCX) [file pntd.0006017.s001.docx]

**Checklist S1. STROBE checklist completed for this manuscript.**

|  | Item No. | Recommendation | Page  No. | Relevant text from manuscript |
| --- | --- | --- | --- | --- |
| **Title and abstract** | 1 | (*a*) Indicate the study’s design with a commonly used term in the title or the abstract | 2 | Line 28 |
|  |  | (*b*) Provide in the abstract an informative and balanced summary of what was done and what was found | 2 | Lines 28-45 |
| Introduction | | | |  |
| Background/rationale | 2 | Explain the scientific background and rationale for the investigation being reported | 5 and 6 | Lines 69-96 |
| Objectives | 3 | State specific objectives, including any prespecified hypotheses | 6 | Lines 96-99 |
| Methods | | | |  |
| Study design | 4 | Present key elements of study design early in the paper | 8 | Lines 131-138 |
| Setting | 5 | Describe the setting, locations, and relevant dates, including periods of recruitment, exposure, follow-up, and data collection | 7 and 8 | Lines 122-128; 131-135; 147-174 |
| Participants | 6 | (*a*) *Cohort study*—Give the eligibility criteria, and the sources and methods of selection of participants. Describe methods of follow-up | NA | NA |
|  |  | (*b*) *Case-control study*—For matched studies, give matching criteria and the number of controls per case | 8 | Lines 131-135 |
| Variables | 7 | Clearly define all outcomes, exposures, predictors, potential confounders, and effect modifiers. Give diagnostic criteria, if applicable | 8, 11 | Lines 131-135; 216-225 |
| Data sources/ measurement | 8* | For each variable of interest, give sources of data and details of methods of assessment (measurement). Describe comparability of assessment methods if there is more than one group | 10 and 11 | Lines 178-210 |
| Bias | 9 | Describe any efforts to address potential sources of bias | 8 and 9 | Lines 137; 174-176 |
| Study size | 10 | Explain how the study size was arrived at | 8 | Lines 141-144 |

Continued on next page

| Quantitative variables | 11 | Explain how quantitative variables were handled in the analyses. If applicable, describe which groupings were chosen and why | 12 | Lines 236-246 |
| --- | --- | --- | --- | --- |
| Statistical methods | 12 | (*a*) Describe all statistical methods, including those used to control for confounding | 12 and 13 | Lines 234-251 |
|  |  | (*b*) Describe any methods used to examine subgroups and interactions | 12 and 13 | Lines 246-251 |
|  |  | (*c*) Explain how missing data were addressed | Not applicable  (NA) | NA |
|  |  | (*d*) *Cohort study*—If applicable, explain how loss to follow-up was addressed | NA | NA |
|  |  | (*e*) Describe any sensitivity analyses | 12 | Lines 243-246 |
| Results | | | | |
| Participants | 13* | (a) Report numbers of individuals at each stage of study—eg numbers potentially eligible, examined for eligibility, confirmed eligible, included in the study, completing follow-up, and analysed | 14 | Lines 258-261 |
|  |  | (b) Give reasons for non-participation at each stage | NA | NA |
|  |  | (c) Consider use of a flow diagram | 14 | Figure 1 |
| Descriptive data | 14* | (a) Give characteristics of study participants (eg demographic, clinical, social) and information on exposures and potential confounders | 14 and 15 | Lines 265-273 |
|  |  | (b) Indicate number of participants with missing data for each variable of interest | NA | NA |
|  |  | (c) *Cohort study*—Summarise follow-up time (eg, average and total amount) | NA | NA |
| Outcome data | 15* | *Cohort study*—Report numbers of outcome events or summary measures over time | NA | NA |
| Main results | 16 | (*a*) Give unadjusted estimates and, if applicable, confounder-adjusted estimates and their precision (eg, 95% confidence interval). Make clear which confounders were adjusted for and why they were included | 19 | 284-305 |
|  |  | (*b*) Report category boundaries when continuous variables were categorized | NA | NA |
|  |  | (*c*) If relevant, consider translating estimates of relative risk into absolute risk for a meaningful time period | NA | NA |

Continued on next page

| Other analyses | 17 | Report other analyses done—eg analyses of subgroups and interactions, and sensitivity analyses | 24 and 25 | Lines 338-369 |
| --- | --- | --- | --- | --- |
| Discussion | | | | |
| Key results | 18 | Summarise key results with reference to study objectives | 26 | Lines 379-385 |
| Limitations | 19 | Discuss limitations of the study, taking into account sources of potential bias or imprecision. Discuss both direction and magnitude of any potential bias | 28 | Lines 442-449 |
| Interpretation | 20 | Give a cautious overall interpretation of results considering objectives, limitations, multiplicity of analyses, results from similar studies, and other relevant evidence | 29 | Lines 450-453 |
| Generalisability | 21 | Discuss the generalisability (external validity) of the study results | 29 | Lines 455-464 |
| Other information | |  | | |
| Funding | 22 | Give the source of funding and the role of the funders for the present study and, if applicable, for the original study on which the present article is based | Online |  |

**Note:** An Explanation and Elaboration article discusses each checklist item and gives methodological background and published examples of transparent reporting. The STROBE checklist is best used in conjunction with this article (freely available on the Web sites of PLoS Medicine at http://www.plosmedicine.org/, Annals of Internal Medicine at http://www.annals.org/, and Epidemiology at http://www.epidem.com/). Information on the STROBE Initiative is available at [www.strobe-statement.org](http://www.strobe-statement.org).
