# Supplementary material for: Schistosoma, other helminth infections, and associated risk factors in preschool-aged children in urban Tanzania
Source: PLoS Negl Trop Dis. 2017 Nov 6;11(11):e0006017. doi: 10.1371/journal.pntd.0006017 (PMC5697890; doi:10.1371/journal.pntd.0006017)
Supplement: S1 Table — (DOCX) [file pntd.0006017.s002.docx]

**S1Table. ADDITIONAL ANALYSIS: RISK FACTORS FOR *S. MANSONI* INFECTION (DEFINED TRACE RESULTS AS POSITIVE BASED ON POINT OF CARE-CIRCULATING CATHODIC ANTIGEN TEST) AMONG 310 UNDER-FIVE CHILDREN IN TEMEKE DISTRICT, DAR ES SALAAM, TANZANIA.**

| **Characteristics** | **All**  **n (%)** | **Crude** | |  | **Adjusted** | |
| --- | --- | --- | --- | --- | --- | --- |
|  |  | **OR (95% CI)** | **p value** |  | **aOR (95% CI)** | **p value** |
| **Age groups (months)** |  |  |  |  |  |  |
| 6-12 | 52 (17) | 1 | 0.3 |  | 1 | 0.2 |
| 13-24 | 92 (30) | 0.78 (0.35-1.72) |  |  | 0.77 (0.35-1.70) |  |
| 25-36 | 71 (23) | 0.76 (0.33-1.74) |  |  | 0.74 (0.32-1.71) |  |
| 37-48 | 57 (18) | 0.78 (0.32-1.88) |  |  | 0.76 (0.31-1.84) |  |
| 49-59 | 38 (12) | 0.50 (0.18-1.41) |  |  | 0.47 (0.16-1.35) |  |
| **Sex** |  |  |  |  |  |  |
| Female | 160 (52) | 1 | 0.9 |  | 1 | 0.9 |
| Male | 150 (48) | 0.97 (0.57-1.66) |  |  | 0.97 (0.56-1.66) |  |
| **Individual deworming history (past 3 months)** |  |  |  |  |  |  |
| Not dewormed | 304 (98) | 1 | 0.9 |  | - | - |
| Dewormed | 6 (2) | 1.10 (0.17-7.23) |  |  | - |  |
| **TB exposure** |  |  |  |  |  |  |
| Unexposed | 121 (39) | 1 | 0.8 |  | - | - |
| Exposed | 189 (61) | 1.08 (0.64-1.84) |  |  | - |  |
| **Number of people in the household** |  |  |  |  |  |  |
| <6 | 190 (61) | 1 | 0.7 |  | - | - |
| ≥6 | 120 (39) | 1.12 (0.65-1.91) |  |  | - |  |
| **Water source** |  |  |  |  |  |  |
| Bore well | 90 (29) | 1 | 0.4 |  | - | - |
| Tap | 153 (49) | 0.42 (0.23-0.75) |  |  | - |  |
| Unknown | 67 (22) | 0.51 (0.25-1.05) |  |  |  |  |
| **Type of household toilet** |  |  |  |  |  |  |
| Pit latrine | 217 (70) | 1 | 0.4 |  | 1 | 0.4 |
| Septic tank | 93 (30) | 1.25 (0.71-2.23) |  |  | 1.30 (0.73-2.33) |  |
| **Hygienic practices^1^** |  |  |  |  |  |  |
| Poor | 36 (12) | 1 | 0.5 |  | 1 | 0.5 |
| Better | 274 (88) | 0.76 (0.34-1.70) |  |  | 0.77 (0.34-1.77) |  |
| **Household income per month (USD)** |  |  |  |  |  |  |
| <100 | 108 (35) | 1 | 0.7 |  | - | - |
| ≥100 | 202 (65) | 0.88 (0.50-1.55) |  |  | - |  |
| **Parent education level** |  |  |  |  |  |  |
| No or primary education | 244 (79) | 1 | 0.5 |  | 1 | 0.5 |
| Secondary/higher education | 66 (21) | 0.80 (0.42-1.55) |  |  | 0.81 (0.41-1.60) |  |
| **Parent occupation** |  |  |  |  |  |  |
| Housewife/unemployed | 196(63) | 1 | 0.2 |  | - | - |
| Employed | 114(37) | 0.70 (0.40-1.20) |  |  | - |  |
| **Family migration history since child birth** |  |  |  |  |  |  |
| No | 188 (61) | 1 | 0.5 |  | - | - |
| Yes | 22 (7) | 0.75 (0.26-2.16) |  |  | - |  |
| Unknown | 99 (32) | 0.83 (0.47-1.48) |  |  | - |  |

^1^ hygiene practices=how often parents wash fruits or vegetable before giving to children;

USD, United States Dollars (1USD=2,190 Tanzanian shillings)

Multivariable model only contained age, sex, type of toilet, hygiene, parent education
