# Supplementary material for: Schistosoma, other helminth infections, and associated risk factors in preschool-aged children in urban Tanzania
Source: PLoS Negl Trop Dis. 2017 Nov 6;11(11):e0006017. doi: 10.1371/journal.pntd.0006017 (PMC5697890; doi:10.1371/journal.pntd.0006017)
Supplement: S2 Table — (DOCX) [file pntd.0006017.s003.docx]

**S2 Table. COMPARISON OF COGNITIVE SCORE AMONG HELMINTH-INFECTED AND NON-INFECTED PRE-SCHOOL-AGED CHILDREN IN DAR ES SALAAM, TANZANIA.**

| **Characteristic** | **All** | **Cognitive score median (IQR)** | | | **p value** |
| --- | --- | --- | --- | --- | --- |
|  | **n (%)** | **Helminth infected**  **n=72** |  | **Helminth non-infected n=230** |  |
| **All** | 310 (100) | 3.22 (2.74-3.44) |  | 3.33 (2.80-3.50) | 0.2 |
| **Age groups (months)** |  |  |  |  | 0.9 |
| 6-12 | 52 (17) | 2.96 (2.70-3.38) |  | 2.99 (2.54-3.39) |  |
| 13-24 | 92 (30) | 2.92 (2.58-3.27) |  | 2.92 (2.72-3.30) |  |
| 25-36 | 71 (23) | 3.22 (2.79-3.44) |  | 3.23 (2.43-3.49) |  |
| 37-48 | 57 (18) | 3.41 (3.38-3.51) |  | 3.50 (3.38-3.62) |  |
| 49-59 | 38 (12) | 3.40 (3.32-3.48) |  | 3.49 (3.40-3.66) |  |
| **Sex** |  |  |  |  | 0.6 |
| Female | 160 (52) | 3.32 (2.85-3.45) |  | 3.32 (2.82-3.50) |  |
| Male | 150 (48) | 3.07 (2.61-3.38) |  | 3.34 (2.77-3.50) |  |
| **Hemoglobin level (g/dl)** |  |  |  |  | 0.9 |
| Anemic <11.0 | 203 (65) | 3.08 (2.68-3.41) |  | 3.21 (2.76-3.48) |  |
| Not anemic ≥11.0 | 104 (34) | 3.39 (3.04-3.48) |  | 3.38 (2.98-3.57) |  |
| Missing | 3 (1) | - |  | - |  |
| **HAZ (z-score≤2)** |  |  |  |  | 0.9 |
| Normal | 243 (78) | 3.33 (2.85-3.45) |  | 3.36 (2.92-3.50) |  |
| Moderate to severe stunted | 67 (22) | 2.85 (2.28-3.14) |  | 2.91 (2.50-3.49) |  |
| **WAZ (z-score≤2)** |  |  |  |  | 0.9 |
| Normal | 228 (74) | 3.31 (2.88-3.44) |  | 3.32 (2.84-3.50) |  |
| Moderate to severe underweight | 82 (26) | 2.91 (2.17-3.42) |  | 3.40 (2.56-3.57) |  |
| **WHZ (z-score≤2)** |  |  |  |  | 0.7 |
| Normal | 225 (73) | 3.07 (2.78-3.43) |  | 3.32 (2.81-3.50) |  |
| Moderate to severe wasted | 79 (25) | 3.33 (2.61-3.45) |  | 3.42 (2.73-3.57) |  |
| Overweight/Obese | 6 (2) | 2.98 (2.57-3.39) |  | 2.95 (2.93-3.01) |  |
| **Deworming status (past 3 months)** |  |  |  |  | 0.9 |
| Not dewormed | 304 (98) | 3.22 (2.70-3.43) |  | 3.33 (2.81-3.50) |  |
| Dewormed | 6 (2) | 3.32 (3.07-3.58) |  | 3.09 (2.44-3.53) |  |
| **Household income per month (USD)** |  |  |  |  | 0.9 |
| <100 | 108 (35) | 3.11 (2.88-3.41) |  | 3.23 (2.76-3.52) |  |
| ≥100 | 202 (65) | 3.31 (2.68-3.45) |  | 3.35 (2.82-3.50) |  |
| **Parent education level** |  |  |  |  | 0.9 |
| No or primary education | 244 (79) | 3.22 (2.78-3.45) |  | 3.34 (2.79-3.50) |  |
| Secondary/higher education | 66 (21) | 3.06 (2.68-3.37) |  | 3.30 (2.86-3.50) |  |
| **Parent occupation** |  |  |  |  | 0.9 |
| Housewife/unemployed | 196(63) | 3.07 (2.64-3.45) |  | 3.24 (2.73-3.49) |  |
| Employed | 114(37) | 3.35 (3.01-3.43) |  | 3.40 (2.91-3.55) |  |
| **Mothers HIV status during pregnancy** |  |  |  |  |  |
| Negative | 256 (82) | 3.28 (2.78-3.44) |  | 3.34 (2.80-3.51) | - |
| Positive | 30 (10) | 2.88 (2.64-3.01) |  | 3.26 (2.84-3.55) |  |
| Unknown | 24 (8) | 1.99 (1.99-1.99) |  | 3.37 (2.70-3.44) |  |
| **Mothers marital status** |  |  |  |  | 0.9 |
| Single | 76 (25) | 2.96 (2.61-3.27) |  | 3.22 (2.73-3.52) |  |
| Married | 217 (70) | 3.33 (2.80-3.44) |  | 3.35 (2.84-3.50) |  |
| Unknown | 17 (5) | 3.29 (2.32-3.58) |  | 3.12 (2.91-3.42) |  |

HAZ, height for age, moderate to severe stunting (z-score≤-2); WAZ, weight for age, moderate to severe underweight (z-score≤-2); WHZ, weight for height, moderate to severe wasting (z-score≤-2): HIV Human immunodeficiency virus
